# Cysteamine Inhibits Glycine Utilisation and Disrupts Virulence in *Pseudomonas aeruginosa*

**DOI:** 10.3389/fcimb.2021.718213

**Published:** 2021-09-22

**Authors:** Douglas J. Fraser-Pitt, Stephen K. Dolan, David Toledo-Aparicio, Jessica G. Hunt, Daniel W. Smith, Niamh Lacy-Roberts, Piumi Sara Nupe Hewage, Teodora N. Stoyanova, Erin Manson, Kevin McClean, Neil F. Inglis, Derry K. Mercer, Deborah A. O’Neil

**Affiliations:** ^1^ NovaBiotics Ltd, Aberdeen, United Kingdom; ^2^ Department of Biochemistry, University of Cambridge, Cambridge, United Kingdom; ^3^ SNIPR Biome, University of Copenhagen, Copenhagen, Denmark; ^4^ School of Pharmacy and Life Sciences, Robert Gordon University, Aberdeen, United Kingdom; ^5^ College of Medical, Veterinary & Life Sciences, University of Glasgow, Glasgow, United Kingdom; ^6^ Proteomics Facility Services, Moredun Research Institute, Penicuik, United Kingdom

**Keywords:** virulence, biofilm, *Pseudomonas aeruginosa*, novel therapeutic, glycine cleavage complex

## Abstract

*Pseudomonas aeruginosa* is a major opportunistic human pathogen which employs a myriad of virulence factors. In people with cystic fibrosis (CF) *P. aeruginosa* frequently colonises the lungs and becomes a chronic infection that evolves to become less virulent over time, but often adapts to favour persistence in the host with alginate-producing mucoid, slow-growing, and antibiotic resistant phenotypes emerging. Cysteamine is an endogenous aminothiol which has been shown to prevent biofilm formation, reduce phenazine production, and potentiate antibiotic activity against *P. aeruginosa*, and has been investigated in clinical trials as an adjunct therapy for pulmonary exacerbations of CF. Here we demonstrate (for the first time in a prokaryote) that cysteamine prevents glycine utilisation by *P. aeruginosa* in common with previously reported activity blocking the glycine cleavage system in human cells. Despite the clear inhibition of glycine metabolism, cysteamine also inhibits hydrogen cyanide (HCN) production by *P. aeruginosa*, suggesting a direct interference in the regulation of virulence factor synthesis. Cysteamine impaired chemotaxis, lowered pyocyanin, pyoverdine and exopolysaccharide production, and reduced the toxicity of *P. aeruginosa* secreted factors in a *Galleria mellonella* infection model. Thus, cysteamine has additional potent anti-virulence properties targeting *P. aeruginosa*, further supporting its therapeutic potential in CF and other infections.

## Introduction

*Pseudomonas aeruginosa* is a versatile and opportunistic human pathogen and often recalcitrant to antibiotic therapy. It is a major cause of ventilator-associated pneumonia (VAP) in hospitals (Koulenti et al., 2009), a less common cause of community acquired pneumonia (CAP) and can also cause infections at other body sites such as wound infections, urinary tract catheter-associated infections and gangrenous diabetic foot ulcer, (Gjødsbol et al., 2006; Nathwani et al., 2014; Trubiano and Padiglione, 2015; Kim et al., 2020). Acute *P. aeruginosa* pneumonia can occur in immunocompetent patients but susceptible and immunocompromised patients such as those receiving mechanical ventilation, or the very young and old are most at risk. Cystic fibrosis (CF) patients have dysfunctional mucocilliary clearance due to thick, dehydrated mucus, and are at risk of lung colonisation by *P. aeruginosa.* Despite recent falls in prevalence, it is still a common species found in the sputum of adults with CF (Cystic Fibrosis Foundation Patient Registry: 2018 Annual Data Report, 2019). This Gram negative motile rod-shaped bacterium was thought to be intrinsically resistant to macrolide antibiotics, though recent evidence suggests that the high minimal inhibitory concentrations (MICs) found in *in vitro* susceptibility testing media were less reflective of antimicrobial activity in physiological conditions (Buyck et al., 2012). Nevertheless, the rising incidence of acquired resistance, particularly to carbapenems has led to its designation as a critical priority pathogen of concern by WHO (World Health Organisation (WHO), 2017).

*P. aeruginosa* can produce a wide array of virulence factors, such as phenazine pigments, as well as cytotoxins, exotoxins, elastase, phospholipase C, and protease A (Azam and Khan, 2019). In CF, opportunistic isolates sometimes establish chronic infection which can be very difficult to eradicate (Lyczak et al., 2000; Gibson et al., 2003). During early infection *P. aeruginosa* isolates are usually highly motile, express type III secretion system effector molecules and the LPS O antigen. Adaptation to chronic infection, often seen within the airways of adults with CF, leads toward a mucoid phenotype, alginate production, acquisition of antibiotic resistance and loss of motility (Smith et al., 2006; Mowat et al., 2011). Evolutionary divergence and adaptation to certain microenvironments can even take place within specific parts of the CF lung (Winstanley et al., 2016). The volatile, and highly toxic compound HCN can be detected in the breath of people with CF colonised with *P. aeruginosa* (Gilchrist et al., 2013; Gilchrist et al., 2015) and its presence in sputum significantly correlates with poorer lung function in individuals with CF and non-CF bronchiectasis (Ryall et al., 2008). Over time, *P. aeruginosa* colonisation is associated with damage to the lung architecture and declining lung function in CF (Kerem et al., 2014). Most of the virulence traits of *P. aeruginosa* are secreted factors under the control of complex, overlapping quorum sensing systems which include those regulated by LasR, RhlR, and MvfR (PqsR) transcriptional regulators. Although these systems have been described as hierarchical (with LasR system at the top) and interdependent (Pesci et al., 1999; McGrath et al., 2004; Heeb et al., 2011), LasR loss of function mutations often arise in chronic CF infections and both RhlR and PQS can function independently (Kostylev et al., 2019).

Cysteamine is a simple aminothiol, produced endogenously through the action of pantetheinases such as vanin-1 in the metabolism of co-enzyme A (Pitari et al., 2000) and is a molecule which may have an underappreciated role in immunity to infection (Fraser-Pitt and Mercer, 2021). It is also a therapeutic licensed for many years for the treatment of the lysosomal storage disorder, cystinosis (Schneider et al., 1995). Cysteamine may also have therapeutic potential as both an oral adjunct to standard of care therapy (SOCT) during infectious exacerbations in CF and as an inhaled maintenance therapy adjunct to antimicrobial therapy. It potentiates antibiotic activity *in vitro* (Charrier et al., 2014; Devereux et al., 2015; Fraser-Pitt et al., 2016; Fraser-Pitt et al., 2018), as well as in murine models of infection (Fraser-Pitt et al., 2018). Oral cysteamine bitartrate, adjunct to SOCT, resulted in statistically significant improvements in patient reported outcome measures and biomarkers of inflammation and infection including reduced white blood cell count, and CRP in patients experiencing an infectious pulmonary exacerbation (Devereux et al., 2020). Cysteamine may also stimulate autophagy, and reduce proteostasis, and some groups have shown that this increases trafficking of the cystic fibrosis transmembrane conductance regulator (CFTR) to the plasma membrane (De Stefano et al., 2014), although other groups have questioned this (Armirotti et al., 2019). Cysteamine has been demonstrated to improve the clearance of intracellular pathogens including *P. aeruginosa* and *Burkholderia cenocepacia* from CF macrophages (Ferrari et al., 2017; Shrestha et al., 2017; Shrestha et al., 2020). Cystic fibrosis has recently seen a paradigm shift in terms of care with the introduction of combinatorial modulator therapies which address the underlying cause of the condition (Middleton et al., 2019). Nonetheless, infectious exacerbations and infection remain as major challenges in CF clinical care and there is still a critical need for novel and effective interventions that target CF symptomology. The broad-spectrum antibiotic potentiation, mucolytic activities, and anti-inflammatory properties of cysteamine may be of benefit to all CF genotypes.

Cysteamine interferes with the REDOX balance of the bacterial cell through conversion to the disulphide cystamine, which depletes intracellular NADPH and cellular reducing power. It can also disrupt the metabolism of *P. aeruginosa*, and it was noted that sub-lethal concentrations of cysteamine could sensitise it to other antimicrobial agents. Cysteamine has a marked effect on pigment production in numerous strains, and the export of pyomelanin from *B. cenocepacia* (Fraser-Pitt et al., 2018). Cysteamine is a highly reactive small molecule, so the precise bacterial cell target (or more likely, targets) that are responsible for such pleiotropic activity remain to be elucidated. As *P. aeruginosa* is a versatile pathogen with a highly flexible metabolism (Dolan et al., 2020), extensive metabolic redundancy can hinder the identification of the precise target of antimicrobials used against this organism. Here we explored further the mechanisms of cysteamine-mediated interference in bacterial metabolism and undermining of virulence traits of *P. aeruginosa.*

Cysteamine has long been known to inhibit the glycine cleavage system found in the mitochondria of eukaryotic cells (Yudkoff et al., 1981) through inhibition of the glycine cleavage system found in the mitochondria (Hayasaka and Tada, 1983). This multienzyme system has a role in glycine and serine catabolism as well as the provision of methyl groups during the biosynthesis of methionine and purines. In mammals the system includes the P-protein, H-protein, L-protein and T-protein - the H-protein is one of the very few lipoylated proteins and the P-protein requires the pyridoxal phosphate (PLP) cofactor. The system is widely evolutionarily conserved in mammalian, plant and many bacterial species. The glycine decarboxylase enzyme is exquisitely sensitive to competitive inhibition by cysteamine with a Ki of 5 µM, which is thought to act *via* interaction with PLP (Lowry et al., 1986). Cysteamine (in common with some other aminothiols) has long been known to react with PLP (Buell and Hansen, 1960) by forming thiazolidine ring. It is known to interfere with PLP-dependent enzyme kinetics (De Marco et al., 1965), and as an endogenous molecule there has been speculation as to the biological role (Terzouli et al., 1998). *P. aeruginosa* possesses two copies of glycine dehydrogenase (decarboxylating) genes, *gcvP1* and *gcvP2*. The GcvP1 and GcvP2 proteins of *P. aeruginosa* are also PLP-dependent enzymes and GcvP2 belongs to the *gcs2* cluster required for glycine catabolism (Lundgren et al., 2013). Glycine is the precursor for HCN production, first demonstrated in *P. fluorescens* (Laville et al., 1998), and the glycine cleavage system has been implicated in HCN production in *P. aeruginosa* too (Sarwar et al., 2016). Inhibition of glycine catabolism caused by increases in ammonia (a product of the reaction) has been reported to increase the abundance of the glycine substrate which in turn raises the amount utilised to make HCN *via* the gene cluster *hcnABC* (Yan et al., 2018).

Here, we demonstrate that cysteamine inhibits glycine utilisation by *P. aeruginosa*, and that the glycine cleavage system has unexpected inhibitory activity on HCN production. We also show for the first time that cysteamine disrupts chemotaxis and swarming motility which may be linked to disruption of metabolism, but the glycine cleavage system is not the only target. One of the most striking impacts of cysteamine treatment on *P. aeruginosa* is the inhibition of pigment production. *Pseudomonas* sp. produce a wide array of pigments, including pyocyanin, a blue REDOX active compound and toxic virulence factor. We have reported qualitative observations on cysteamine-mediated changes in pigment production on a wide range of *P. aeruginosa* strains previously (Fraser-Pitt et al., 2018) and here we quantify the effects on pyocyanin. *P. aeruginosa* also secretes the fluorescent green-yellow iron-chelating siderophore, pyoverdine. Pyoverdine is necessary for virulence in numerous infection models and chemical inhibitors of the production of this siderophore have demonstrated potential in mouse models of infection (Kang et al., 2019) therefore we also investigated the impact of cysteamine on pyoverdine fluorescence. Whilst inhibition of biofilm formation by cysteamine has been demonstrated previously against a wide range of bacteria (Charrier et al., 2014; Fraser-Pitt et al., 2016; Fraser-Pitt et al,. 2018), here we demonstrate that it can reduce exopolysaccharide production in an alginate-producing strain and inhibits the induction of biofilm formation by the aminoglycoside, tobramycin. These data illustrate that cysteamine can dysregulate multiple aspects of *P. aeruginosa* virulence.

## Materials and Methods

Unless otherwise stated, chemicals, reagents and media were purchased from Sigma-Aldrich (MO, USA).

### Bacterial Strains, Growth Media and Culture Conditions

*P. aeruginosa* PAO1 a commonly used type strain (Holloway, 1955) was purchased from the American Type Culture Collection. PA14 (UCBPP-PA14) is a clinical burns isolate (Rahme et al., 1995) first sequenced in 2004 (He et al., 2004) and representative of common clonal isolates found worldwide in numerous disease states including CF (Wiehlmann et al., 2007). NH57388A is a stable *mucA* mucoid isolate derived from CF sputum which produces pyocyanin (Norman et al., 2016) and NH57388B is a non-mucoid quorum-sensing deficient strain that does not produce N-acylhomoserine lactone signalling molecules (Hoffmann et al., 2005).

The Δ*gcvP2* PAO1 mutant was created by PCR amplifying flanking regions 800-1000 bp upstream and downstream of the *gcvP2* ORF. Upstream and downstream regions were then overlapped and cloned into the suicide vector pEX19Gm using Gibson assembly as described previously (Huang and Wilks, 2017). The resulting deletion plasmid was then transformed into *P. aeruginosa* PAO1 by electroporation and selected for on LB plates containing 50 µg/ml gentamycin. Deletion mutants were identified *via sacB* mediated sucrose counter-selection and confirmed by PCR. Primers used are described in **Table S1**.

Growth media included cation-adjusted Müller-Hinton broth 2 (MHB). M9 minimal media was prepared (Miller, 1972) with 20 mM carbon sources as described previously (Sarwar et al., 2016). Growth was assessed by optical density (O.D.) of 100 µl volumes at 625 nm over time at 37°C in 96-well flat-bottomed plates (Thermo-Fisher, MA, USA) using a Synergy (Biotek, VT, USA) plate reader. When not in routine use strains were cryopreserved in 20% glycerol (80% Müller-Hinton broth) at -80°C for long term storage.

### Susceptibility (MIC) and Chemotaxis Experiments

The MIC (minimum inhibitory concentration at which 100% of bacterial growth is prevented) of cysteamine in different media was calculated as described using the CLSI broth microdilution procedure M07-A10 (Wayne, 2015). The standard solid media used to maintain strains was Müller-Hinton agar (MHA) containing 1.5% granulated agar (Melford Laboratories, UK), Chemotaxis swimming and swarming assessments were performed on M9 minimal media containing 0.3% granulated agar, and 20 mM glucose, or 0.2% w/v casamino acids +/- cysteamine at 200 mg/L in 2-compartment plates. Overnight cultures grown in MHB were pelleted, washed and resuspended in M9 minimal salts to achieve uniform OD600 = 0.4 ± 0.02 and 2 µl volumes were spotted onto the plate surface in a sterile class II containment hood prior to incubation at 37°C (Parales and Ditty, 2018). Images were captured at 24 and 48 h using a Nikon D5100 (Japan) at the same conditions and settings (f/5.3, 1/50 s, ISO-1600) and a Samsung Galaxy S20 (Samsung, South Korea) for colour images to demonstrate inhibition in pigment production. Colony area was calculated from colony radii. The experiment was conducted on three occasions.

### Determination of Glycine in Culture Supernatants and Bacterial Cell Lysates

Bacterial cultures were prepared in Müller Hinton broth of each strain (PAO1 parent, and Δ*gcvP2* strain) using standardised inoculums of 1 in 150 dilution of 0.5 McFarland standard from overnight cultures. Three 10 ml cultures of each strain were used as controls (no exposure to cysteamine) and 3 cultures were treated with 200 mg/L cysteamine. After 24 h at 37°C aliquots of bacterial cultures were serially diluted to determine final cfu/ml and then pelleted at 5,000 g for 5 min. Clarified supernatants were carefully removed without disturbing the pellets and filter sterilised using syringe-driven 0.22 µM PES filter and stored at -80°C prior to analysis. Bacterial pellets were washed 1 x in ice-cold PBS and centrifuged again before being resuspended in 1 ml sterile distilled water and transferred to a heating block and heated at 90°C to lyse bacteria. Lysates were also stored at -80°C prior to analysis. Glycine concentrations were determined fluorometrically using a glycine assay kit ab211000 (Abcam, Cambridge UK) according to the manufacturer’s instructions on a black-walled 96-well microtitre plate and read using Biotek Synergy plate-reader (Biotek, VT, USA). Uninoculated Müller-Hinton broth (both treated and untreated) were used as controls and the addition of cysteamine did not affect the assay or the concentration of glycine in the media which was 2871.42 ±121.63 ng/ml (standard deviation).

### Qualitative Hydrogen Cyanide Detection

Volatile HCN production was determined qualitatively *via* colorimetric detection using Whatman ashless 125 mm diameter filter paper discs (Whatman, UK) cut to fit above the surface of a two-compartment culture dish and impregnated with a solution containing 5 mg of copper (II) ethylacetoacetate and 5 mg 4,4’methylenebis-(N,N-dimethylaniline) in chloroform (Castric and Castric, 1983). Discs were left to dry and stored at 4°C before use. Bacterial lawns were prepared from overnight cultures resuspended to a uniform 0.5 McFarland cell density and seeded onto two-compartment plates containing MHA or MHA with 200 mg/L cysteamine and allowed to dry in a class II containment hood. HCN detection discs were overlain above both compartments and sterile modified microfuge tube lids were used to keep the discs from touching the culture surface and to prevent condensation on the lids. Plates were incubated upright at 37°C. PAO1, and Δ*gcvP2* PAO1 cultures tended to produce HCN quicker and were examined 18 h post inoculation, whereas PA14, and NH57388A and B were examined at 24 h. Experiments were conducted on three occasions.

### *C. elegans* Assessment of HCN Mediated Paralysis

*C. elegans* was maintained under standard conditions (Wood, 1988). Worms were synchronised to achieve a population of young adults using the bleaching method described by Stiernagle (Stiernagle, 2006).

Bacteria were grown overnight on MHA, cultures were then diluted 1:100 in MHA and spread onto new MHA plates with or without the incorporation of cysteamine at the sub-inhibitory concentrations indicated in the agar and cultured for a further 24 h at 37°C. Bacterial lawns were overlain with nitrocellulose membranes in a method adapted from Darby (Darby et al., 1999). Synchronized worms were then transferred to the nitrocellulose membrane and incubated at room temperature for 6 h. Worms were carefully lifted from the hydrophobic nitrocellulose membrane by 100 µl M9 buffer and transferred to optically clear 96-well flat-bottomed plates to be examined for paralysis and scored for survival every hour, for 6 hours, by inspection under an Axiovert 40 CFL Microscope (Zeiss, Germany) at x 100 magnification. Worm paralysis was defined as no movement after mechanical stimulation.

### Quantification of Pyocyanin

Pyocyanin was extracted from 12 ml *P. aeruginosa* cultures grown statically in MHB for 24 hours at 37°C following inoculation with standardised 1:150 dilutions (80 µl) of 0.5 McFarland suspensions of bacterial cells +/- cysteamine at stated concentrations. Following incubation bacterial cells were removed by centrifugation at 5,000 g for 5 min. Supernatants were then transferred to a fresh tube and further clarified by passing through a 0.22 µM PES filter. Pyocyanin was extracted *via* liquid phase partitioning first with chloroform and then again with 0.2 N HCl and quantified by measuring the absorbance of 100 µl volumes on 96-well flat-bottomed plates at 520 nm using a Synergy plate reader, concentrations were calculated using the extinction coefficient 17.072 (Kurachi, 1958; Essar et al., 1990).

### Quantification of Exopolysaccharide Production

The effect of cysteamine on the production of exopolysaccharide by the stable mucoid *P. aeruginosa* NH57388A was compared with the non-mucoid AHL-deficient NH57388B strain. Exopolysaccharide production was determined in a method adapted from that devised by Ramus (1977). Each 12 ml culture was inoculated with standardised 1:150 dilutions (80 µl) of 0.5 McFarland suspensions of bacterial cells from overnight cultures on MHA plates and cysteamine was added to final concentrations of 2 - 200 mg/L. Cultures were incubated statically for 120 h at 37°C. Following incubation bacterial cells were removed by centrifugation at 5,000 g for 5 min at 4°C, 1 ml volumes were transferred to sterile microfuge tubes and 100 µl of acidified Alcian blue (1 mg/ml) was added which precipitates polyanionic exopolysaccharides such as alginate. Tubes were briefly vortexed (10 sec) to mix before further centrifugation at 5,000 g for 5 min at 4°C which pellets the precipitate. Aliquots of supernatant (100 µl) were then transferred to clear 96-well flat-bottomed plate and absorbance recorded at 610 nm using a Synergy plate reader. EPS concentration is inversely proportionate to absorbance of the unbound dye, and samples were quantified by comparison to a standard curve prepared using known concentrations of sodium alginate.

### Biofilm Assay

**Figure 4:**
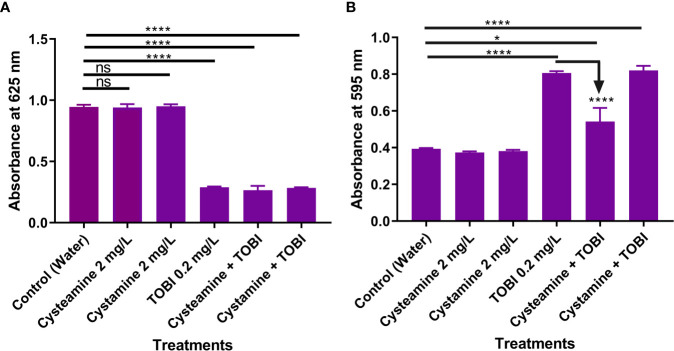
Cysteamine and cystamine do not inhibit growth of planktonic cells **(A)** at 2 mg/L. Whilst sub-lethal concentrations of tobramycin (TOBI) reduced the number of planktonic cells at 24 h **(A)** induced biofilm formation over the same time period **(B)** and this is significantly reduced by adjunct treatment with cysteamine but not the disulfide, cystamine (n=9). ****p ≤ 0.0001 one-way ANOVA followed by Dunnet’s *post-hoc* analysis. ns, not significant. *p is less than or equal to 0.05.

Attachment of *P. aeruginosa* Pa14 biomass to 6-well plates was measured using an adapted crystal violet biofilm assay (O’Toole et al., 1999) using standardised inoculum and MHB growth media ± cysteamine and/or tobramycin as described in **Figure 4**. Planktonic growth in culture supernatants was measured after 24 h at 37°C by transferring 100 µl of each replicate to optically clear microtitre plates and recording absorbance at 625 nm. Crystal violet was released from washed surface-attached cells using 100% ethanol and 100 µl of each replicate transferred to microtitre plates quantified at 595 nm.

### Fractionation of *P. aeruginosa* Whole Cell Proteome Liquid Chromatography and Electrospray Ionisation Tandem Mass Spectrometry (LC-ESI-MS/MS) Analysis

#### Sample Preparation and Separation by SDS PAGE

Six separate 10 ml cultures of *P. aeruginosa* PAO1 in MHB were prepared in sterile Universal tubes using single colonies grown from frozen stock revived overnight on MHA plates. Cultures were incubated statically for 20 hours and diluted in fresh media if necessary, to achieve growing cultures with an optical density of 0.3 at 625 nm. Each culture was then sub-cultured 1 ml into 9 ml of fresh MHB and incubated for 4 hours at 37°C to achieve mid-logarithmic growth. Bacterial cells were pelleted by centrifugation at 5,000 g for 5 min. Following this, three of these cultures were resuspended in MHB media alone and three were resuspended in media containing sub-inhibitory 250 mg/L cysteamine and all cultures were incubated for a further 4 hours at 37°C. Bacterial cells were then pelleted by centrifugation at 5,000 g for 5 min at 4°C and washed once with ice cold PBS. Bacterial cell pellets were resuspended and lysed in 200 µl of BugBuster (Merck Millipore, MA, USA) containing a protease inhibitor cocktail (Complete Mini, Roche Diagnostics GmbH, Basel, Switzerland) at the manufacturer’s recommended concentration. Lysate protein concentrations were determined by bicinchoninic acid (BCA) assay (Smith et al., 1985) and equal amounts 5 mg total protein of whole cell lysates were separated by one-dimensional sodium dodecyl sulfate polyacrylamide gel electrophoresis (SDS-PAGE) using a precast 4-20% gradient Tris-glycine gel. Gel was visualised with Coomassie based EZBlue (Sigma, MO, USA) stain.

#### LC-ESI-MS/MS Analysis

Each of 6 lanes of whole cell proteins was fractionated into 21 slices. Each slice was subjected to in-gel tryptic digestion, reduction, alkylation and trypsinolysis, followed by reversed-phase liquid chromatographic separation of tryptic peptides using rapid monolithic column chromatography and ESI-MS/MS using a fast-scanning three-dimensional ion trap tandem mass spectrometer (Bruker Daltonics amaZon-ETD).

LC was performed using an Ultimate 3000 nano-HPLC system (Dionex, LC-Packings) using the method described previously (Burgess et al., 2009).

Mass spectrum peak lists for peptides and fragmentation ions were compared with *in-silico* published sequence for *P. aeruginosa* PAO1 utilising the Mascot™ V2.4.1 (Matrix Science) search engine. Mascot search parameters were set in accordance with published guidelines (Taylor and Goodlett, 2005) and to this end, fixed (carbamidomethyl “C”) and variable (oxidation “M” and deamidation “N,Q”) modifications were selected along with peptide (MS) and secondary fragmentation (MS/MS) tolerance values of 0.5Da whilst allowing for a single 13C isotope. Protein identifications obtained from each of the 21 individual gel slices per lane were compiled using the Meta-score protein compilation feature within the ProteinScape bioinformatics platform (Chamrad et al., 2003). From the compiled protein lists individual protein identifications were inspected manually and considered significant only if a) two peptides were matched for each protein, b) peptides were represented by a sequence coverage of >5% and c) each matched peptide contained an unbroken “b” or “y” ion series represented by a minimum of four contiguous amino acid residues. The identification or absence of a protein in each treatment condition is only a semi-quantitative indication of relative abundance of proteins and individual leads need to be confirmed with further analysis.

### Preparation of Sterile *P. aeruginosa* Conditioned Media

*P. aeruginosa* NH57388A (A) was cultured for 24 h at 37°C in the presence of sub-inhibitory concentrations of cysteamine at 0, 2, 10, 20, 100 and 200 mg/L in cation-adjusted MHB (Thermo-Fisher, MA, USA). Culture supernatants were then clarified by centrifugation at 5,000 g for 5 min and then filter-sterilised by passing through a 0.22 µM syringe-driven PES filter.

### Assessment of the Toxicity of Secreted *P. aeruginosa* Factors Produced in Conditioned Media or Sputum in *Galleria mellonella*

All *Galleria mellonella* larvae were purchased from Live Food UK Ltd (Rooks Bridge, UK) and, upon arrival, kept in wood shavings at 20°C in the dark for 3 days prior to challenge. Larvae with approximate weight of 0.25–0.30 g, which were not already melanized were assigned randomly to the different challenge groups or unmanipulated or vehicle control cohorts. Larvae were surface-sterilised with 70% ethanol. Ten microlitre volumes of vehicle control, or conditioned media was injected into the last left proleg as appropriate for each treatment using a 100 microlitre Hamilton syringe which was sterilized with ethanol and larvae were placed into separate sterile petri dishes at 37°C. A melanisation score was determined by observation over time according to criteria described by Kay et al. (2019), adapted from Senior et al. (2011). Insects were considered dead when they become unresponsive to physical stimuli and turned black in colour. The toxicity of *P. aeruginosa* conditioned media was assessed with 10 larvae per treatment group. Survival was plotted using Kaplan-Meier survival distribution method and tested for statistical significance using Log-rank (Mantel-Cox) test.

### Statistical Analysis

All statistical analysis was performed using GraphPad Prism 9.0.0. software using appropriate tests outlined in in the methods section above or in the figure legend.

## Results

### Cysteamine Inhibited the Utilisation of Glycine in *Pseudomonas aeruginosa*

**Figure 1:**
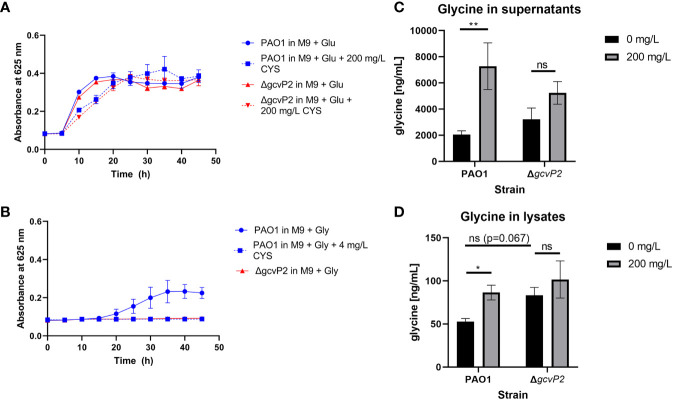
**(A)** *P. aeruginosa* PAO1 and Δ*gcvP2* growth over time at 37°C in M9 minimal media supplemented with 20 mM glucose with or without the addition of 200 mg/L cysteamine (n=3). **(B)** Only the PAO1 parent strain can grow in M9 supplemented with 20 mM glycine as the sole carbon source and 4 mg/L cysteamine prevents growth of the parent strain on this carbon source (n=3). **(C)** Glycine concentrations in bacterial culture supernatants grown for 24 h in cation-adjusted Müller-Hinton broth at 37°C with or without the addition of 200 mg/L cysteamine (n=3). Glycine levels in media alone were unaffected by cysteamine and were calculated as 2871.42 ±121.63 ng/ml (standard deviation). **p ≤ 0.01, two-way ANOVA with Tukey’s test. ns, not significant **(D)** Glycine concentrations in bacterial lysates from cultures grown for 24 h in cation-adjusted Müller-Hinton broth at 37°C with or without the addition of 200 mg/L cysteamine (n=3). *p ≤ 0.05, Two-way ANOVA with Tukey’s test. ns, not significant.

Cysteamine has a high minimal inhibitory concentration (MIC) of 500 mg/L in glucose minimal media against *P. aeruginosa* strains (Fraser-Pitt et al., 2018) and **Table S2**. Here we show that 200 mg/L has little effect on growth in either the Δ*gcvP2* mutant or PAO1 parent strain (**Figure 1A**). However, cysteamine prevented the utilisation of glycine as a sole carbon source by *P. aeruginosa* PAO1 (**Figure 1B**) as treatment with as little as 4 mg/L cysteamine completely prevented growth. As the *gcs2* cluster is responsible for glycine utilisation in *P. aeruginosa* and glycine decarboxylase is the target in mammalian cells we characterised the growth of Δ*gcvP2* mutant and PAO1 parent strain in glucose and glycine minimal media. As expected Δ*gcvP2* did not grow in glycine minimal media. The addition of high sub-inhibitory concentrations of cysteamine (200 mg/L) to growing cultures of the PAO1 parent and Δ*gcvP2* strains in Müller-Hinton broth caused glycine to accumulate in both the supernatant (**Figure 1C**) and bacterial lysates (**Figure 1D**), but significant increases were only seen in the parent strain. Levels of glycine, in cysteamine-treated supernatants only, were above those of uninoculated Müller-Hinton broth, indicating that glycine is created during metabolism in this media but that it does not ordinarily accumulate. The Δ*gcvP2* strain had higher mean concentrations of glycine in the untreated pellets than the parent strain, though this was not significant (p=0.067).

### Electrospray Ionisation Tandem Mass Spectrometry Proteome Analysis of *P. aeruginosa* Exposed to Cysteamine

Whole cell proteomic analysis was conducted to examine the effect of cysteamine-mediated blockade on glycine utilisation by sub-inhibitory concentrations of cysteamine.

365 proteins were confidently identified by ESI tandem MS according to criteria outlined above. Fifteen proteins were found only in cysteamine-treated conditions across all replicates, and 66 proteins were confidently identified only in control conditions. The remaining 284 were confidently identified in both conditions. **Table 1** lists proteins confidently identified only in cysteamine treated *P. aeruginosa* cells and **Table 2** shows proteins only confidently identified in control conditions.

**Table 1:** Proteins confidently identified only in the cysteamine-treated *P. aeruginosa* PAO1 cells according to criteria outlined by Taylor and Goodlett, 2005.

Treatment condition	Accession number	Protein name	Locus tag	Gene name	Function/pathway
**Cysteamine treated**	NP_249916.1	FAD-dependent NADPH:quinone reductase	PA1225	mdaB	Oxidative stress
	NP_251132.1	glycine cleavage system protein T2	PA2442	gcvT2	Glycine cleavage
	NP_252418.1	hypothetical protein	PA3729		Uncharacterised
	NP_253999.1	aldehyde dehydrogenase	PA5312	pauC	Polyamine catabolism
	NP_251721.1	hypothetical protein	PA3031		Uncharacterised
	NP_253256.1	GTPase ObgE	PA4566	obgE	Response to stimulus
	NP_254244.1	ATP synthase F0F1 subunit delta	PA5557	atpH	ATP synthesis coupled proton transport
	NP_249578.1	acetyl-CoA synthetase	PA0887	acsA	Acetate utilisation
	NP_254101.1	hypothetical protein	PA5414		Uncharacterised
	NP_251872.1	6-phosphogluconolactonase	PA3182	pgl	Pentose phosphate pathway
	NP_250981.1	glucose-sensitive porin	PA2291	oprB2/opbA	Glucose uptake
	NP_253007.1	hypothetical protein	PA4317		Uncharacterised
	NP_249540.1	thioredoxin reductase	PA0849	trxB2	Removal of superoxide radicals
	NP_251134.1	serine hydroxymethyltransferase	PA2444	glyA2	Glycine biosynthesis, tetrahydrofolate interconversion
	NP_251992.1	hypothetical protein	PA3302		Uncharacterised
	NP_251148.1	hypothetical protein	PA2458		Uncharacterised

**Table 2:** Proteins confidently identified only in the control *P. aeruginosa* PAO1 cells according to criteria outlined by Taylor and Goodlett, 2005.

Treatment condition	Accession number	Protein name	Locus tag	Gene name	Function/pathway
**Control**	NP_252997.1	chemotactic transducer	PA4307	pctC	Chemotaxis, response to amino acid, signal transduction
	NP_252999.1	chemotactic transducer	PA4309	pctA	Chemotaxis, response to amino acid, signal transduction
	NP_254241.1	ATP synthase F0F1 subunit beta	PA5554	atpD	Produces ATP from ADP in the presence of a proton gradient across the membrane. The catalytic sites are hosted primarily by the beta subunits.
	NP_253259.1	octaprenyl-diphosphate synthase	PA4569	ispB	Isoprenoid synthesis
	NP_064729.1	glycyl-tRNA synthetase subunit alpha	PA0009	glyQ	Translation. Transfer of glycine amino acid to nascent peptides
	NP_250378.1	spermidine synthase	PA1687	speE	Polyamine synthesis
	NP_250243.1	cytochrome C oxidase cbb3-type subunit	PA1552	ccoP1	Electron transport chain
	NP_248735.1	hypothetical protein	PA0045	csgG	Uncharacterised
	NP_252294.1	response regulator	PA3604	erdR	Ethanol detoxification
	NP_251020.1	hypothetical protein	PA2330		Uncharacterised
	NP_252750.1	thioredoxin	PA4061	YbbN	Glycerol ether metabolic process, cell redox homeostasis
	NP_251251.1	chemotaxis transducer	PA2561	ctpH	Chemotaxis, detection of phosphate ion, signal transduction
	NP_249783.1	flagellin type B	PA1092	fliC	Swimming motility
	NP_249688.1	PqsB protein	PA0997	pqsB	Quorum sensing
	NP_253277.1	cytochrome C551 peroxidase	PA4587	ccpR	Oxidation-reduction processes
	NP_254114.1	alcohol dehydrogenase	PA5427	adhA	Ethanol detoxification
	NP_253754.1	phosphoribosyl-ATP pyrophosphatase	PA5067	hisE	Histidine biosynthesis
	NP_251985.1	HIT family protein	PA3295		Uncharacterised
	NP_253563.1	OsmE family transcriptional regulator	PA4876	osmE	Response to stimulus
	NP_249102.1	twitching motility protein	PA0411	pilJ	Signal transduction, chemotaxis, pilin biosynthesis
	NP_252082.1	nitrous-oxide reductase	PA3392	nosZ	Denitrification pathway
	NP_250368.1	hypothetical protein	PA1677		Uncharacterised
	NP_248992.1	polyamine transporter	PA0301	spuE	Polyamine transport
	NP_253335.1	uracil phosphoribosyltransferase	PA4646	upp	UMP biosynthesis *via* salvage pathway
	NP_253991.1	D-amino acid dehydrogenase small subunit	PA5304	dadA	D-amino acid metabolic process, phenylalinine metabolic process
	NP_252548.1	carboxylesterase	PA3859		Carboxylic ester hydrolase activity
	NP_248816.1	hypothetical protein	PA0126		Uncharacterised
	NP_252508.1	hypothetical protein	PA3819		Rick_17kDa_Anti domain-containing protein
	NP_251263.1	chemotaxis transducer	PA2573		Signal transduction
	NP_252331.1	amino acid permease	PA3641		Sodium/alanine symporter
	NP_253795.1	hypothetical protein	PA5108		Uncharacterised
	NP_249700.1	hypothetical protein	PA1009		Uncharacterised
	NP_249342.1	indole-3-glycerol phosphate synthase	PA0651	trpC	Tryptophan biosynthesis/metabolism
	NP_253323.1	chemotaxis transducer	PA4633		Signal transduction
	NP_253619.1	50S ribosomal protein L9	PA4932		Translation
	NP_253821.1	carboxyl-terminal protease	PA5134	ctpA	Cell envelope organisation, cell wall biogenesis, pathogenesis, protein secretion by the type III secretion system, signal transduction
	NP_252016.1	ATP-dependent Clp protease proteolytic subunit	PA3326	clpP2	Proteolysis
	NP_251816.1	heat-shock protein IbpA	PA3126	ibpA	Protein folding
	NP_250219.1	cell division protein	PA1528	zipA	Division septum assembly, FtsZ-dependent cytokinesis
	NP_253376.1	ferric iron-binding periplasmic protein	PA4687	hitA	Iron ion homeostasis
	NP_252347.1	methionine aminopeptidase	PA3657	map	Cellular protein metabolic process
	NP_253430.1	tRNA pseudouridine synthase B	PA4742	truB	RNA metabolic processes
	NP_251543.1	outer membrane lipoprotein	PA2853	oprI	Outer membrane protein
	NP_253085.1	nucleotide-binding protein	PA4395	yajQ	Unknown
	NP_253132.1	bifunctional sulfate adenylyltransferase subunit 1/adenylylsulfate kinase	PA4442	cysNC	hydrogen sulfide biosynthesis
	NP_250493.1	ATP-dependent protease ATP-binding subunit ClpX	PA1802	clpX	Protein folding
	NP_251342.1	chemotaxis transducer	PA2652		Cellular response to an organic substance, chemotaxis
	NP_250759.1	carbamoyl transferase	PA2069		Biosynthetic processes, nodulation
	NP_250734.1	hypothetical protein	PA2044		Uncharacterised
	NP_249345.1	S-adenosylmethionine decarboxylase	PA0654	speD	Spermidine biosynthetic process
	NP_250439.1	enoyl-CoA hydratase	PA1748		
	NP_249090.1	cystathionine beta-synthase	PA0399		Cysteine biosynthetic process from serine
	NP_248924.1	transcriptional regulator	PA0233		Regulation of transcription
	NP_249020.1	hypothetical protein	PA0329		Uncharacterised
	NP_253705.1	peptide methionine sulfoxide reductase	PA5018	msrA/pmsR	Cellular response to oxidative stress
	NP_253559.1	hypothetical protein	PA4872		Uncharacterised
	NP_250270.1	hypothetical protein	PA1579		Uncharacterised
	NP_251947.1	periplasmic tail-specific protease	PA3257	algO/prc	Signal transduction, proteolysis
	NP_253977.1	hypothetical protein	PA5290	yifB	Uncharacterised
	NP_253012.1	hypothetical protein	PA4322		AAA domain-containing protein, ATPase activity, ATP binding
	NP_251853.1	cytidylate kinase	PA3163	cmk	Nucleobase-containing small molecule interconversion, pyrimidine nucleotide metabolic process
	NP_254112.1	phosphoribosylaminoimidazole carboxylase ATPase subunit	PA5425	purK	IMP biosynthesis *via de novo* pathway
	NP_249563.1	phenylalanine 4-monooxygenase	PA0872	phhA	Tyrosine biosynthesis, L-phenylalanine catabolism
	NP_253137.1	histidinol-phosphate aminotransferase	PA4447	hisC1	L-histidine biosynthesis
	NP_252221.1	bacterioferritin	PA3531	bfrB	Intracellular sequestering of iron
	NP_250946.1	paerucumarin biosynthesis protein PvcC	PA2256	pvcC	Iron chelation
	NP_249644.1	thioredoxin	PA0953	helX (DsbE)	Antioxidant activity, oxidoreductase activity

Although the glycine dehydrogenase (decarboxylating) proteins encoded by *gcvP1* and *gcvP2* were not amongst the proteins detected with confidence in either cysteamine treated or controls, the neighbouring serine hydroxymethyltransferase (GlyA2) and glycine cleavage system protein T2 (GcvT2) were only confidently detected in cysteamine treated cells, suggesting an increased protein abundance in this condition. The glycyl tRNA synthetase (*glyQ*) was amongst the proteins found only in the control condition.

Also of interest, the FAD-dependent NADPH:quinone reductase, MdaB (modulator of drug activity B), was identified only in the cysteamine treated condition. This has been implicated in the detoxification of certain compounds and it is linked to quinone and REDOX cycling (Adams et al., 2005). It is not known if this is involved in a shift in NADP : NADPH ratio previously reported to be induced by cysteamine (Fraser-Pitt et al., 2018).

**Figure 2:**
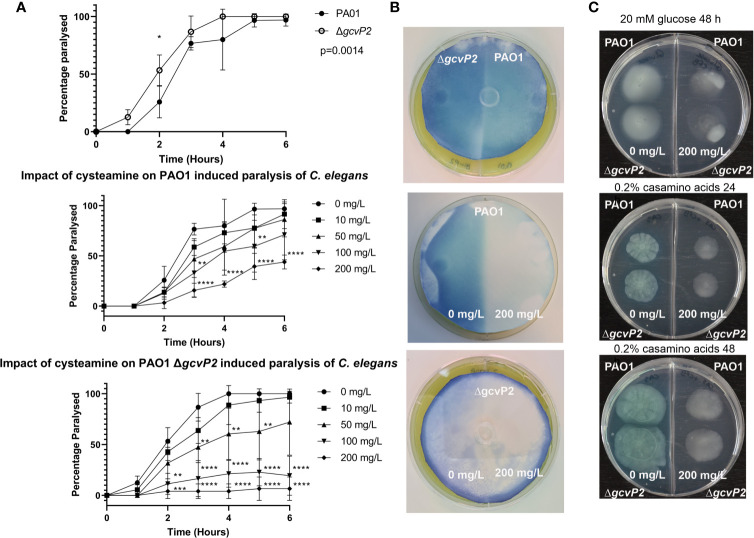
*C. elegans* paralysis assay, HCN production, and chemotaxis and motility assays. **(A)** Both the PAO1 parent strain and the Δ*gcvP2* mutant induced paralysis in *C. elegans* over time. The Δ*gcvP2* mutant produced significantly higher levels of paralysis than the parent strain. Cysteamine provided dose-dependent protection from paralysis in both mutant and parent strain, suggesting cysteamine inhibited the HCN produced by *P. aeruginosa* cultures. *p ≤ 0.05, **p ≤ 0.01, ***p ≤ 0.001, ****p ≤ 0.0001 two-way ANOVA and Šidák’s multiple comparisons test. **(B)** Colorimetric detection of volatile HCN produced by *P. aeruginosa* PAO1 and ΔgcvP2 mutant grown for the same time (n=3) showed that the mutant liberated more HCN. Experiments on 2-compartment agar plates with either MHA alone (-) or supplemented (+) with sub-inhibitory 200 mg/L cysteamine showed that HCN production in both the mutant and the parent strain were inhibited by cysteamine. **(C)** Chemotactic swimming motility of both PAO1 and the ΔgcvP2 mutant on 0.3% M9 agar containing 20 mM glucose was inhibited by the addition of 200 mg/L cysteamine at 48 h, as demonstrated by the abolition of concentric rings. Swarming motility, as defined by dendritic outgrowth, was also inhibited on 0.3% agar containing 0.2% w/v casamino acids at 24 h and 48 h, and the effect on pigment production is also evident (Representative images from n=3).

Chemotaxis proteins PctA, PctC, CtpH, PA4633 and PA2652 were amongst those identified only in control conditions. Flagellin (the structural unit of the bacterial flagellum) and the twitching motility protein PilJ, were also only detected in the control condition. This provided the rationale to investigate the potential for cysteamine to inhibit chemotaxis and chemotactic swimming and swarming motility (**Figure 2**).

### Cysteamine Inhibits HCN Production

Glycine is converted into HCN by *P. aeruginosa* by the contiguous HCN synthase genes *hcnABC.* A blockade of glycine cleavage by cysteamine in *P. aeruginosa* might be expected to raise glycine levels, as it does when administered to rats (Iwama et al., 1997). Mutation in the *gcsR* gene, a TyrR-like enhancer-binding protein of the alternative sigma factor RpoN (σ^54^) which regulates the glycine cleavage system in *P. aeruginosa* PAO1 (Sarwar et al., 2016), was demonstrated to enhance HCN-dependent paralytic-killing in the *Caenorhabditis elegans* model. HCN production is also known to be under the control of the hierarchical quorum sensing system in *P. aeruginosa*.

We examined the effects of cysteamine on the production of HCN by the Δ*gcvP2* mutant and the parent PAO1 We did this by comparing the ability of PAO1 and Δ*gcvP2* to induce paralysis in a *C. elegans* model (Darby et al., 1999), and examined any effect of the incorporation of cysteamine into the growth media on the ability of each strain to induce paralysis (**Figure 2A**). We also examined HCN production directly by incorporating sub-inhibitory concentrations of cysteamine into agar plates seeded with lawns of each isolate and detecting volatile HCN produced during culture over time (**Figure 2B**).

Despite effective blockade of glycine utilisation by cysteamine in *P. aeruginosa* at 4 mg/L (**Figure 1B**), the incorporation of cysteamine (at 200 mg/L) into MHA plates surprisingly inhibits the production of HCN, despite having no effect on bacterial growth. The Δ*gcvP2* mutant produced more HCN than the PAO1 parent strain over the same incubation time, and induced paralysis in *C. elegans* quicker than the parent strain, probably due to increased substrate availability. Cysteamine also dose-dependently protected *C. elegans* from paralysis (**Figures 2A, B**).

Cysteamine also had a marked effect on chemotaxis on semi-solid (0.3% agar) media containing glucose as the sole carbon source. Radial growth was apparent in control conditions at 48 h for both the parent strain and Δ*gcvP2* and both were impacted by the incorporation of cysteamine into the growth media, as surface growth out from the central inoculum was reduced and disorganised. When grown on 0.2% w/v casamino acids medium there is evidence of swarming motility, indicated by dendritic outgrowth from the inoculum, in both mutant and parent strain which is inhibited by cysteamine. The impact on pigment production was also particularly evident when grown on casamino acids (**Figure 2C**). The inclusion of cysteamine also impacted on the area of growth, with significant reductions in growth area for both the parent strain and Δ*gcvP2* mutant on agar containing 0.2% w/v casamino acids at 24 and 48 h. Cysteamine reductions in growth on 20 mM glucose were less evident at 24 h and only significant for the Δ*gcvP2* mutant at 48 h (**Figure S1**).

### Inhibition of Exopolysaccharide Production

**Figure 3:**
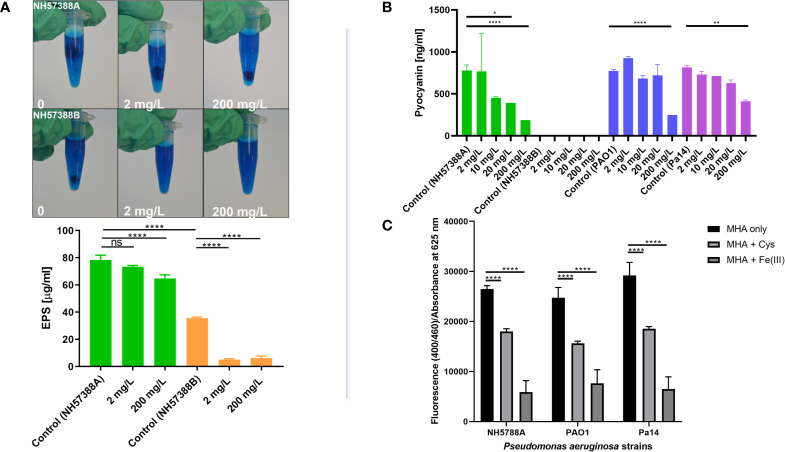
Cysteamine inhibits exopolysaccharide, pyoverdine and pyocyanin production. **(A)** Exopolysaccharide precipitated from sterile-filtered MHB culture supernatants of *P. aeruginosa* strains NH57388A and NH57388B with acidified Alcian blue following culture for 48 h at 37°C. Quantification of exopolysaccharide secreted into culture supernatant by NH57388A mucoid strain treated with cysteamine at 2 and 200 mg/L for 48 h at 37°C. ****p ≤ 0.0001 one-way ANOVA and Dunnet’s multiple comparison test, ns, not significant (n=3). Cysteamine had a dose-dependent inhibitory effect on exopolysaccharide secreted from mucoid NH57388A and non-mucoid quorum sensing deficient NH57388B strains. **(B)** Cysteamine-mediated inhibition of pyocyanin production by *P. aeruginosa* strains PAO1, PA14 and NH57388A following 20 h treatment at 37°C with the concentration range shown. *p ≤ 0.05, **p ≤ 0.01, ****p = < 0.0001, One-way ANOVA and Tukey’s test (n=3). **(C)** Inhibition of pyoverdine expression after 20 h growth in MHB by cysteamine at 200 mg/L in multiple strains as detected by fluorescence at 400/460 ex/em normalised to absorbance at 625 nm and compared with quenching induced by excess iron (1 mM Fe(III)Cl3). ****p = < 0.0001, Two-way ANOVA and Tukey’s test (n=3).

Exopolysaccharide (EPS) production is important for biofilm formation which is critical for *P. aeruginosa* chronic infection of the CF lung (Høiby, Ciofu and Bjarnsholt, 2010), at least three exopolysaccharides contribute toward biofilm production, including Psl, Pel and alginate (Reviewed by Ghafoor et al., 2011). Cysteamine significantly inhibited the amount of EPS secreted by mucoid *P. aeruginosa* strain NH57388A into (and precipitated from) culture media and, as expected, the non-mucoid quorum sensing mutant NH57388B produced significantly less exopolysaccharide than NH57388A (**Figure 3A**). Cysteamine had a significant effect on the exopolysaccharide secreted by both the alginate-producing NH57388A strain and the non-mucoid NH57388B at high sub-inhibitory concentrations.

### Cysteamine Inhibits the Production of Pyocyanin and Pyoverdine

Here we quantify the effect of a range of cysteamine doses on pyocyanin (**Figure 3B**) secreted by *P. aeruginosa* PAO1 type strain, NH57388A alginate-producing strain and PA14 clinical strains grown in MHB. We also demonstrate that sub-inhibitory concentrations of cysteamine inhibit pyoverdine fluorescence (**Figure 3C**).

### Cysteamine Reduces Tobramycin-Mediated Biofilm Induction

Aminoglycosides are routinely used for the control of *P. aeruginosa* in CF patients, including short-term parenteral administration during pulmonary exacerbations and chronic use as inhalation therapies. The tobramycin inhalation powder is safe, efficacious (Geller et al., 2011; Konstan et al., 2011) and has been adopted for routine chronic use for patients colonised with *P. aeruginosa* for some time, mainly owing to superior convenience, and therefore improved patient compliance, over nebulised therapies (Blasi et al., 2018). Tobramycin inhalation is known to improve lung function over time, and reduce the exacerbation rate (Smith et al., 2018). At the same time exposure to sub-inhibitory concentrations of tobramycin is known to induce biofilm formation by *P. aeruginosa* (Hoffman et al., 2005) including in CF isolates, independent of minimum inhibitory concentration (MIC) and resistance status (Elliott et al., 2010). RpoN is known to play a role in tobramycin tolerance and biofilm formation (Lloyd et al., 2017; Viducic et al., 2017; Shao et al., 2018).

Here we examined the effect of combining sub-inhibitory concentrations of cysteamine and tobramycin together on the attachment of *P. aeruginosa* clinical strain PA14 to polystyrene plates using the modified crystal violet method (O’Toole, et al, 1999). Although sub-inhibitory concentrations of some other antibiotics can induce biofilm formation, tobramycin was chosen due to its use as an inhalation maintenance therapy in CF, and PA14 was selected due to clinical relevance. Tobramycin impaired planktonic growth at 0.2 mg/L but encouraged biofilm formation, whereas cysteamine at 2 mg/L had no impact on planktonic growth at all, alone or in combination with tobramycin at this concentration. However, the combination of tobramycin and cysteamine significantly reduced the biofilm formation (**Figure 4**). The disulfide, cystamine, had no effect on biofilm formation at 24 h.

### Cysteamine Inhibited *P. aeruginosa* Virulence in *G. mellonella* Model

We have already demonstrated potentiation of ciprofloxacin and tobramycin against *P. aeruginosa* in different murine infection models (Fraser-Pitt et al., 2018), but the degree to which inhibition of bacterial virulence factor production contributed to enhanced antimicrobial efficacy is difficult to assess and was not examined at the time. The larvae of the wax moth *G. mellonella* have been developed as a non-mammalian *in vivo* infection model which has now been used extensively to test antimicrobial agents. *P. aeruginosa* has strain-dependent virulence in this model, which correlates well to virulence in mice (Jander et al., 2000). Although some *P. aeruginosa* virulence mechanisms are contact-dependent, many are secreted, so we have assessed the toxicity of sterile-filtered culture supernatants derived from *P. aeruginosa* strains at equal cell densities, treated with different sub-inhibitory concentrations of cysteamine for 20 h at 37°C prior to injection into the insect haemolymph. The lethality of *Pseudomonas* infection in *G. mellonella* can be extreme and varies widely between strains (Cullen et al., 2015). Therefore, we decided to examine sterile-filtered supernatants from cultures of *P. aeruginosa*, using the less virulent mucoid strain, NH57388A

**Figure 5:**
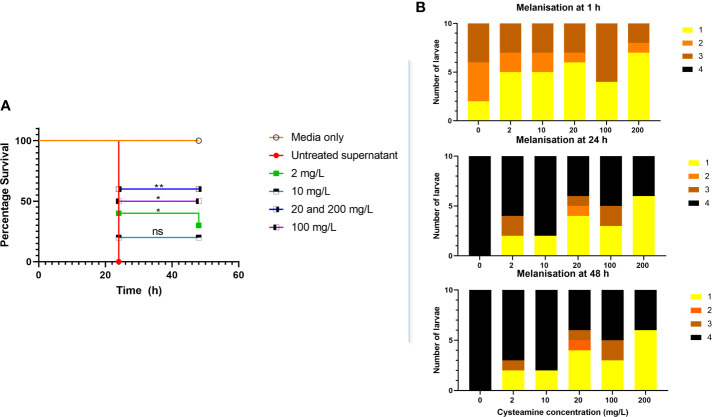
**(A)** Cysteamine inhibited the toxicity of microbial secreted factors toward* G. mellonella* produced in culture. *P. aeruginosa* NH57388A was cultured for 24 h at 37°C in the presence of cysteamine at 0, 2, 10, 20, 100 and 200 mg/L and 10 µl volumes of sterile-filtered supernatants were injected into *G. mellonella* larvae and survival recorded over 48 h. *p ≤ 0.05, **p ≤ 0.01 log-rank (Mantel-Cox) test, ns, not significant. **(B)** Melanisation in response to *P. aeruginosa* secreted factors was rapid as illustrated by the change in melanisation score over time and inhibited by cysteamine. Scoring criteria developed by Kay et al. (2019) are as follows: 1. No signs of melanisation, 2. Signs of nodulation and lateral line melanisation, 3. Systemic melanisation (>50%), 4. Deceased (no movement for 20 min) with full systemic melanisation).

Exposure to 2, 20, 100 and 200 mg/L cysteamine significantly reduced the toxicity of sterile-filtered supernatants of NH57388A in *G. mellonella* (**Figure 5**) and reduced the rapid melanisation seen at 1 h post injection.

## Discussion

Cysteamine was found to inhibit multiple aspects of *P. aeruginosa* virulence building upon previous data demonstrating antibiofilm activity and the ability to reduce pigment production. Results presented here demonstrate for the first time that cysteamine can inhibit glycine utilisation in prokaryotic cells, but the links between this metabolic disruption and virulence factor production are not known. We also demonstrate inhibition of HCN and exopolysaccharide production and that cysteamine (at therapeutically achievable doses) hinders biofilm formation induced by tobramycin.

### Inhibition of Glycine Utilisation and Links to Virulence

Cysteamine inhibits the evolutionary well-conserved glycine cleavage system in mammalian cells. This system is found on the inner membrane of mitochondria in mammalian cells and is composed of H, P, L and T proteins. It has been reported that cysteamine increases the Km for glycine and V_max_ for the P-protein (Hayasaka and Tada, 1983), and is reported to prevent both glycine oxidation and synthesis (Yudkoff et al., 1981). *P. aeruginosa* PAO1 encodes two copies of *gcvH*, *gcvP* and *gcvT* genes and has three copies of the serine hydroxymethyltransferase gene *glyA* which converts glycine to serine and plays an important role in the generation of one carbon units for methylation as well as (together with serine dehydratase) conversion to pyruvate for utilisation as a sole carbon (or nitrogen) source (Sarwar et al., 2016). The inability to utilise glycine in the presence of small concentrations of cysteamine (**Figure 1**) could be indicative of a similar blockade.

Inhibition of glycine cleavage could be expected to interfere with bacterial C1 metabolism and methylation. The relative abundance of methyl-accepting chemotaxis proteins (MCPs) may be reduced by cysteamine, with the proteins CtpH, PctA, and PctB are amongst those identified by proteomics only in control conditions (**Table 2**). The same chemotaxis proteins have been implicated in virulence by Sheng et al., 2019, which demonstrate inhibition of these MCPs (plus CtpM and others) by overexpression of MapZ, a c-di-GMP-dependent inhibitor of methylation of MCPs protected mice in an intraperitoneal model of acute infection. Generation of the second messenger c-di-GMP is directly linked to the glycine cleavage system *via* serine hydroxymethyltransferase (ShrA/GlyA2) in *P. aeruginosa* (Pu et al., 2018) and high levels of c-di-GMP are linked to chronic virulence traits including exopolysaccharide production (Lee et al., 2007; Chung et al., 2008), alginate polymerisation and export *via* Alg44 (Amikam and Galperin, 2006; Merighi et al., 2007; Oglesby et al., 2008), biofilm formation (Hickman et al., 2005), and antibiotic efflux (Nicastro et al., 2014; Poudyal and Sauer, 2018; Wang et al., 2018).

In the proteomics data GcvT2 and GlyA2 were only identified in the cysteamine-treated condition (**Table 1**). In combination with a lack of detectable glycyl tRNA synthetase (GlyQ) in cysteamine treated conditions, it is tempting to speculate that these alterations are a direct feedback response to the inhibition of glycine decarboxylation, and the corresponding increase in glycine abundance (**Table 2**). Whilst we confirmed cysteamine has impacts on swimming and swarming chemotactic behaviours (**Figure 2**) there was an equal effect on both the Δ*gcvP2* and parent strain. *P. aeruginosa* possesses two glycine cleavage operons therefore it could be that any impact on chemotaxis in the Δ*gcvP2* mutant is compensated by secondary activity of *gcvP1*, or that cysteamine disrupts chemotaxis *via* a different mechanism. The measurement of glycine in bacterial lysates and supernatants confirmed cysteamine-mediated disruption of glycine utilisation as glycine was increased in cysteamine treated PAO1 cells (**Figures 1C, D**). Although the increase was only significant in the parent strain, mean increases in glycine were seen in the mutant also, which may suggest the *gcvP1* has residual activity impaired by cysteamine treatment and this is worthy of future investigation.

The glycine cleavage system was initially linked to the production of pyocyanin *via* the TyrR-like enhancer-binding protein (EBP) GcsR (PA2449) believed to regulate the *gsc2* operon, which includes *gcvH2, gcvP2, glyA2, sdaA*, and *gcvT2* encoding some of the proteins mentioned above found in cysteamine-treated cells (Lundgren et al., 2013). However, this was subsequently disproved by the same group who determined the inhibition of pyocyanin in the initial study was due to unrelated mutations. This study confirmed the EBP nature of GcsR which enhances the transcriptional activity of RpoN upstream of the *gsc2* operon. As discussed above, the Δ*gscR* strain increased the paralytic killing of *C. elegans* postulated to be caused by diverting glycine into HCN production, although the authors did not look at HCN production directly and saw no changes in expression of *hcnABC* genes (Sarwar et al., 2016). Our study shows the Δ*gcvP2* strain does indeed produce more HCN than the parent strain, confirming increased substrate availability can drive increases in *hcnABC* conversion of glycine to HCN. It is therefore surprising that we see cysteamine-mediated decreases in HCN production and reduced paralysis in *C. elegans*, (**Figure 2**) and reduced virulence in the *G. mellonella* model (**Figure 5**) despite inhibition of glycine catabolism.

**Figure 6:**
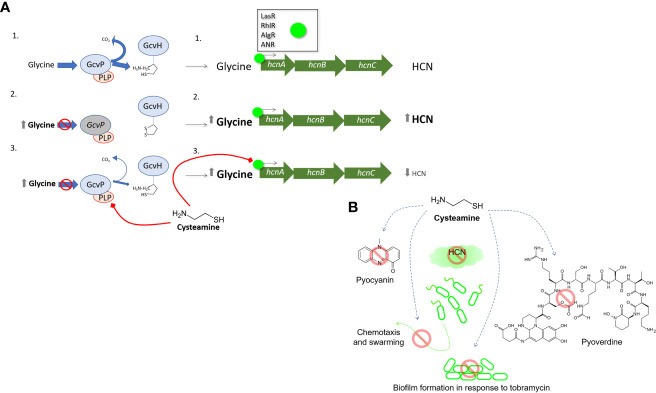
The effect of cysteamine on HCN production **(A)** provides clues to the anti-virulence activities of cysteamine. In untreated *P. aeruginosa* 1. Glycine cleavage is uninterrupted and normal levels of glycine are available as precursors for *hcnABC* gene cluster for the generation of HCN which is under varying levels of regulatory control by LasR, RhlR, AlgR, MvfR and ANR. In scenario 2. inactivation of glycine decarboxylase (GcvP2) prevents glycine utilisation, and higher concentrations of glycine are available for the synthesis of HCN by HcnABC and an increase of HCN is detected. In scenario 3. cysteamine reversibly inactivates GcvP2 *via* thiazolidine formation with the cofactor PLP. More glycine is available for HCN synthesis, but less HCN synthesis occurs, indicating cysteamine also impairs the regulation of one or more of the regulators listed above. Panel **(B)** summarises the anti-virulence activities reported in this manuscript.

Transcriptional control of *hcnABC*, which converts glycine into HCN and CO_2_, is complex (**Figure 6**). HCN formation is greater under low oxygen tension and the anaerobic regulator of arginine deiminase and nitrate reductase (ANR) protein is one of a few transcriptional regulators which influence *hcnABC* (Zimmermann et al., 1991; Laville et al., 1998). ANR, and the *E. coli* homologue FNR, sense oxygen *via* an iron-sulfur cluster, and DNA binding is maximal under reducing conditions (Ye et al., 1995; Lazazzera et al., 1996), and iron availability also impacts HCN expression *via* ANR which is abolished in *P. fluorescens* when iron is depleted (Blumer and Haas, 2000). The quorum sensing response activators LasR and RhlR also regulate HCN production in *P. aeruginosa* (Latifi et al., 1995; Winson et al., 1995; Reimmann et al., 1997; Whiteley et al¸1999) and can act in concert to maximise HCN production (Pessi and Haas, 2000). The GacA two-component system *via* two regulatory RNAs, RsmZ and RsmY, also influences HCN expression indirectly *via* quorum sensing due to enhanced expression of *rhlI*, or directly post-transcriptionally, by enhancing the translation of *hcnABC* (Pessi and Haas, 2001).

Therefore, we have uncovered that cysteamine blocks the glycine cleavage system and this should, in theory, increase HCN production as shown with the Δ*gcvP2* mutant. Instead, cysteamine must also simultaneously act upon one or more of the *P. aeruginosa* global regulatory systems to impair HCN production, despite the presumed backup of cellular glycine. These data highlight that precursor availability is a key cue in HCN production in *P. aeruginosa*, yet a cue which is still subordinate to the additional regulatory mechanisms detailed above. Thus, the dissection of a single, core metabolic target of cysteamine has unexpectedly shed light into the regulatory hierarchy of HCN production in *P. aeruginosa* – a vital virulence factor in this organism.

### Inhibition of Multiple Virulence Factors by Cysteamine

Quorum sensing systems coordinate the production of various virulence factors in *P. aeruginosa* including the production of phenazines. Our previous work demonstrated inhibition of pigment production in multiple *P. aeruginosa* strains and inhibition of the export of the pigment pyomelanin in *B. cenocepacia* strains (Fraser-Pitt et al., 2018). Here we used the chloroform extraction method to confirm the dose-dependent reduction in pyocyanin production by *P. aeruginosa* in response to cysteamine. Similarly, cysteamine interferes with the production of the fluorescent, iron-chelating pyoverdine virulence factor. Pyoverdine production has been described as essential for virulence in *in vivo* models both with mutants of type strains (Meyer et al., 1996) and in CF isolates (Kang et al., 2019).

Anecdotal evidence from the CARE-CF-1 trial led us to also examine the effect of cysteamine on exopolysaccharide production. In the trial, a dominant pyomelanin-producing sputum isolate of *P. aeruginosa* was mucoid at day 0, (before treatment) and non-mucoid at day 14 following exposure to cysteamine. It is already known that the thiol, N-acetylcysteine (already used as a mucolytic treatment in CF), can reduce extracellular polysaccharide production in various bacteria (Olofsson et al., 2003). Cysteamine also demonstrated a significant, though modest, reduction in secreted exopolysaccharide produced by the mucoid alginate-producing NH57388A strain.

The anti-biofilm activity of cysteamine has been reported before. High concentrations can disrupt biofilms, whereas sub-MIC concentrations can prevent biofilms by *P. aeruginosa* and *B. cepacia* complex strains (Charrier et al., 2014; Fraser-Pitt et al., 2016; Fraser-Pitt et al., 2018). Here we demonstrate that concentrations of cysteamine that are therapeutically achievable *via* systemic dosing can also reduce the biofilm promoting activity of another agent, tobramycin. This antibiotic is frequently employed prophylactically in adults with CF known to be colonised with *P. aeruginosa* to suppress their growth, but sub-lethal concentrations of the antibiotic have long been known to encourage biofilm formation (Hoffman et al., 2005). A recent study (Tahrioui et al., 2019) demonstrated the adaptive response mechanism is thought to involve extracellular DNA release, PrrF small regulatory RNAs and quorum sensing *via* the pseudomonas quinolone signal (PQS).

The results above show cysteamine was capable of disrupting metabolism and a number of secreted virulence factors in *P. aeruginosa.* This was confirmed using the *G. mellonella* invertebrate model. Larvae melanised rapidly in response to *P. aeruginosa* NH57388A supernatants and died within 24 h. Cysteamine provided some protection against this toxic response and the results were evident quickly due to the effect on reducing melanisation at 1 h. Most deaths in the larvae injected with supernatants from treated *P. aeruginosa* occurred at 24 h with few thereafter indicating a protection from toxicity.

In conclusion, cysteamine inhibits glycine utilisation and dysregulates virulence factor production in *P. aeruginosa* at sub-inhibitory concentrations and reduces the secretion of toxic factors by *P. aeruginosa*. The strategic development of novel therapeutics which have anti-virulence properties and operate differently to conventional antibiotics has been described as essential for our progress in combating antimicrobial resistance (Fleitas Martínez et al., 2019). Compounds which can improve the effectiveness of other agents and at the same time reduce virulence factor production at sub-lethal concentrations are less likely to drive the emergence of resistance (Vale et al., 2016; Munguia and Nizet, 2017). As an endogenously produced molecule, expressed at sites of inflammation and infection, it maybe that cysteamine has an underappreciated role in innate defence against infection. In common with many innate immune effectors, it may work best in concert with other agents, particularly at therapeutic doses where the effects on individual virulence factors (except for the reduction in tobramycin mediated biofilm induction) are less easy to demonstrate. However, the ability to reduce several virulence factors at once in this versatile pathogen likely means the whole effect is greater than the sum of its parts. There is clearly more than one bacterial cell target for cysteamine and given the common effects on eukaryotic and prokaryotic glycine cleavage systems, cysteamine will affect both the host and the pathogen. Critical regulatory systems in *P. aeruginosa* are clearly disrupted by cysteamine and a systems-based approach may be useful in further understanding the impact of this aminothiol on microbial virulence and metabolism. The positive impact of cysteamine on several virulence factors in this still-common CF pathogen combined with the multivalent properties of this molecule (mucolysis, antibiotic-potentiation etc.) add to the potential utility for this endogenous molecule as a therapeutic in CF, and potentially beyond as we continue the search for agents to tackle the antimicrobial resistance crisis.

## Data Availability Statement

The original contributions presented in the study are publicly available. This data can be found here: [https://www.ebi.ac.uk/pride/archive/projects/PXD027156].

## Author Contributions

DF-P is the corresponding author and wrote the original draft and submitted manuscript, had roles in supervision, conceptualisation, investigation, data curation and analysis of the project. SD made significant contributions to the conceptualisation of the project and was involved in investigation and analysis of data and reviewing/editing of the manuscript. DT-A is an investigating scientist and was involved in generating data and analysis and reviewing/editing. JH was involved in *C. elegans* investigation and data analysis. DS has made significant contributions to conceptualisation of the project and in *G. mellonella* method development. NL-R was a visiting student involved in investigation, data curation and analysis. PN was a visiting student involved in investigation, data curation and analysis. TS provided expertise for *C. elegans* method development and the review/editing of the manuscript. EM provided mass spectrometry expertise, and was involved in investigation and data generation. KM provided mass spectrometry expertise and was involved in investigation and data generation, reviewing/editing. NI supervised proteomics investigation. DM conceptualisation, review/editing. DO supervision, conceptualisation, review/editing. All authors contributed to the article and approved the submitted version.

## Funding

We would like to thank the Society for Applied Microbiology (Sfam) for funding the proteomics work contained in this manuscript *via* a New Lecturer Award (awarded to DF-P whilst a Lecturer at Edinburgh Napier University) in 2011. NL-R and PN had student placements at NovaBiotics Ltd and were generously supported by Equate Scotland Careerwise programme for women in STEM. SD was generously supported by a BBSRC Flexible Talent Mobility Account 2020-2021. All other work was commercially funded by NovaBiotics Ltd. NovaBiotics Ltd provided support in the form of salaries for authors DF-P, DT-A, JH, DS, DM, and DO.

## Conflict of Interest

DF-P, DT-A, JH, DS, and DM are all employees of NovaBiotics Ltd. DAO is the CEO, CSO and a shareholder of NovaBiotics Ltd. DF-P and DO are authors of patent; WO2016198842A1, IL256162D0 - An amino thiol for use in the treatment of an infection caused by the bacterium mycobacterium spp. DO is also author of the following patents; US9364491B2 (and other territories) - Antimicrobial compositions with cysteamine/A composition comprising an antibiotic and a dispersant; WO2016046524A1 - Use of cysteamine in treating infections caused by yeasts/moulds; US9782423B2 (and other territories) - Antibiotic compositions comprising an antibiotic agent and cysteamine; US9339525B2 (and other territories) - Inhibition of biofilm organisms; and US20190175501A1 pending (and other territories) - Microparticles comprising a sulphur-containing compound. This does not alter our adherence to Frontiers policies on sharing data and materials.

The remaining authors declare that the research was conducted in the absence of any commercial or financial relationships that could be construed as a potential conflict of interest.

## Publisher’s Note

All claims expressed in this article are solely those of the authors and do not necessarily represent those of their affiliated organizations, or those of the publisher, the editors and the reviewers. Any product that may be evaluated in this article, or claim that may be made by its manufacturer, is not guaranteed or endorsed by the publisher.
