# Supplementary figures and images for: Cysteamine Inhibits Glycine Utilisation and Disrupts Virulence in Pseudomonas aeruginosa
Source: Front Cell Infect Microbiol. 2021 Sep 22;11:718213. doi: 10.3389/fcimb.2021.718213 (PMC8494450; doi:10.3389/fcimb.2021.718213)

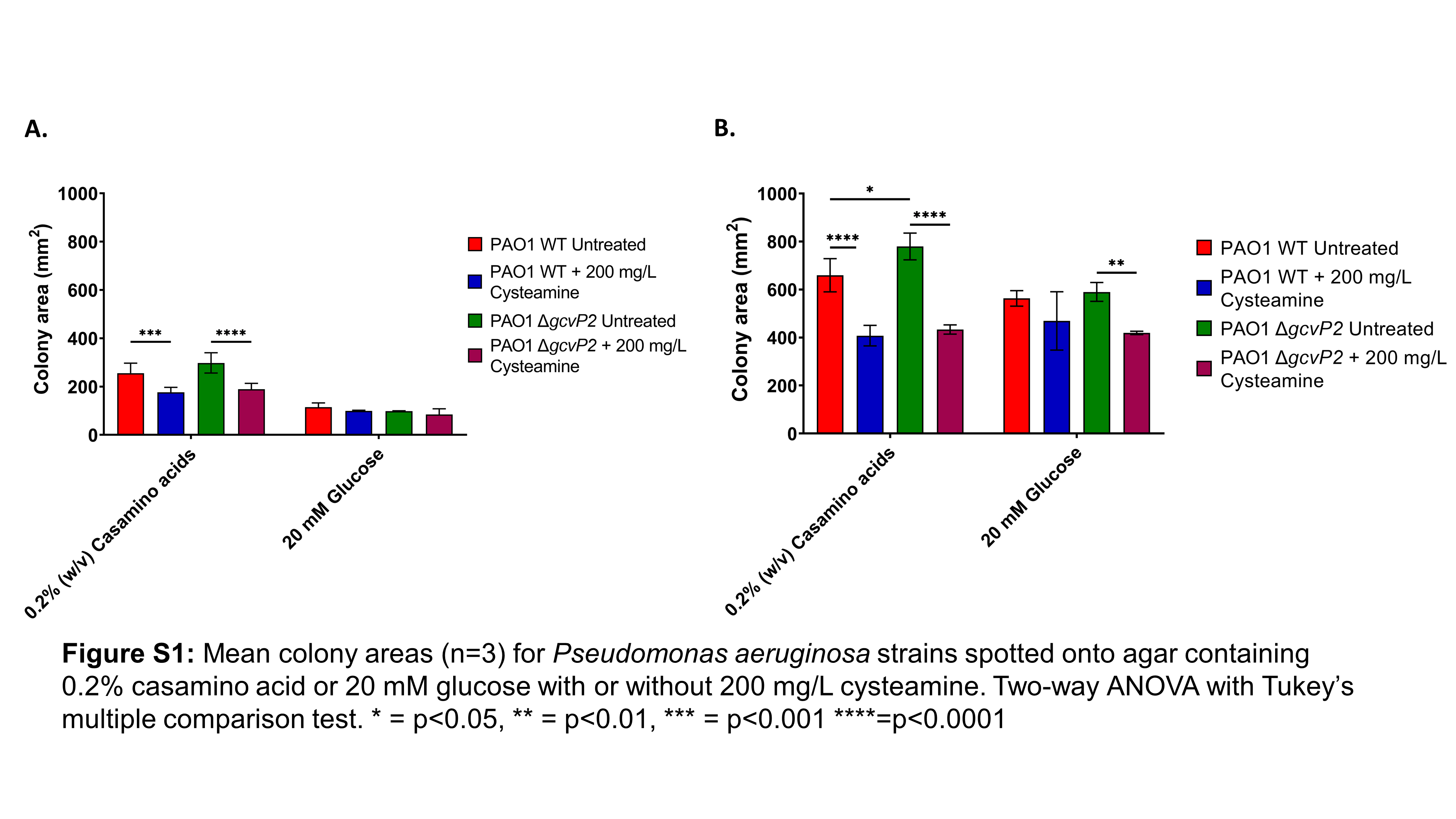

Supplement: Supplementary file 1 [file Image_1.tif]
