# Supplementary material for: Cysteamine Inhibits Glycine Utilisation and Disrupts Virulence in Pseudomonas aeruginosa
Source: Front Cell Infect Microbiol. 2021 Sep 22;11:718213. doi: 10.3389/fcimb.2021.718213 (PMC8494450; doi:10.3389/fcimb.2021.718213)
Supplement: Supplementary file 2 [file Table_1.docx]

**Supplementary Information**

| Primer | Sequence |
| --- | --- |
| gcvP2 PAO1 UP F | acccggggatcctctCGACAACTACGACTTCCTG |
| gcvP2 PAO1 UP R | cgtcactccCCGAGGTTCAGGTTATCG |
| gcvP2 PAO1 DW F | cctcggGGAGTGACGGACGAGGAG |
| gcvP2 PAO1 DW R | ctgcaggtcgactctAATACCGCGGAGTTGAGC |

Table S1: Primers used in this study
