# Supplementary material for: Cysteamine Inhibits Glycine Utilisation and Disrupts Virulence in Pseudomonas aeruginosa
Source: Front Cell Infect Microbiol. 2021 Sep 22;11:718213. doi: 10.3389/fcimb.2021.718213 (PMC8494450; doi:10.3389/fcimb.2021.718213)
Supplement: Supplementary file 3 [file Table_2.docx]

**Supplementary Information**

| Species | Strain | MIC for cysteamine in Mueller Hinton Broth 2 (mg/L) | MIC for cysteamine in 20mM glucose M9 media (mg/L) |
| --- | --- | --- | --- |
| *Pseudomonas aeruginosa* | PAO1 | 250 | 500 |
| *Pseudomonas aeruginosa* | Δ*gcvP2* | 250 | 500 |
| *Pseudomonas aeruginosa* | Pa14 | 500 | 500 |
| *Pseudomonas aeruginosa* | NH57388A | 250 | 500 |
| *Pseudomonas aeruginosa* | NH57388B | 250 | 500 |

Table S2: MIC for cysteamine against each of the strains used in this manuscript
